# Red Blood Cell-Derived Extracellular Vesicles: An Overview of Current Research Progress, Challenges, and Opportunities

**DOI:** 10.3390/biomedicines11102798

**Published:** 2023-10-16

**Authors:** Si-Rui Ma, Hou-Fu Xia, Ping Gong, Zi-Li Yu

**Affiliations:** 1 State Key Laboratory of Oral & Maxillofacial Reconstruction and Regeneration, Key Laboratory of Oral Biomedicine Ministry of Education, Hubei Key Laboratory of Stomatology, School & Hospital of Stomatology, Wuhan University, Wuhan 430079, China; masirui@whu.edu.cn (S.-R.M.); xiahoufw@whu.edu.cn (H.-F.X.); 2 Department of Oral and Maxillofacial Surgery, School and Hospital of Stomatology, Wuhan University, Wuhan 430079, China; 3 Department of Anesthesiology, School and Hospital of Stomatology, Wuhan University, Wuhan 430079, China

**Keywords:** red blood cells, extracellular vesicles, diseases, biomarker, delivery vectors

## Abstract

Red blood cell-derived extracellular vesicles (RBC EVs) are small, spherical fragments released from red blood cells. These vesicles, similar to EVs derived from other cell types, are crucial for intercellular communication processes and have been implicated in various physiological and pathological processes. The diagnostic and therapeutic potential of RBC EVs has garnered increasing attention in recent years, revealing their valuable role in the field of medicine. In this review, we aim to provide a comprehensive analysis of the current research status of RBC EVs. We summarize existing studies and highlight the progress made in understanding the characteristics and functions of RBC EVs, with a particular focus on their biological roles in different diseases. We also discuss their potential utility as diagnostic and prognostic biomarkers in diseases and as vectors for drug delivery. Furthermore, we emphasize the need for further research to achieve selective purification of RBC EVs and unravel their heterogeneity, which will allow for a deeper understanding of their diverse functions and exploration of their potential applications in diagnostics and therapeutics.

## 1. Introduction

Currently, it is widely accepted that extracellular vesicles (EVs) are spherical fragments of cell membranes released from various cell types under physiological as well as pathological conditions. As we searched the literature, we determined that EVs of red blood cell origin were first identified in 1987 by Johnstone et al. from the culture media of sheep reticulocytes [1]. At the time, the released vesicles were named exosomes. Since then, a lot of studies have revealed that exosomes and EVs, in general, can be released by many other cell types [2,3,4]. Although many studies have demonstrated that exosomes and/or EVs can play critical roles in the development of various diseases, particularly cancer, few studies have evaluated the function of red blood cell-derived EVs (RBC EVs) in cancer [3,5]. RBC EVs are produced by the most abundant cell type in the blood, but their function remains elusive [6]. To date, most studies have been focused on the release of RBC EVs from stored RBCs and their role in transfusion-related adverse reactions because transfusion represents the most widely used therapeutic approach to save a person’s life [7]. EVs are released both by endogenous circulating RBCs and RBCs during storage [7,8]. And an increasing number of studies are gradually uncovering the characteristics of RBC EVs released by endogenous circulating RBCs and their roles in various diseases. RBC EVs have been recognized as significant contributors to thrombosis, hemostasis, infectious diseases, cancer, and inflammation [8,9,10,11].

Furthermore, elevated levels of RBC EVs have been observed in the bloodstream of individuals with various pathological conditions, including sickle cell disease, thrombosis, cardiovascular diseases, and glucose-6-phosphate deficiency (G6PD) [9,12]. Therefore, the concentration and cargo profiles of RBC EVs have been put forward as a potential biomarker for the diagnosis of pathological states or diseases [8,9]. Due to their natural ability to transmit their cargoes between cells, RBC EVs possess many favorable properties, such as stability, biocompatibility, low immunogenicity, and biological barrier permeability [13]. RBC EVs are recommended when we develop EV-based delivery systems with longer lifetimes in circulation [13]. And RBC EVs have been developed as delivery vectors of RNA, siRNA, and immRNA to fight against various diseases, including cancer [13,14,15].

In this review, we provided a brief summary of the biogenesis, cargo profiles, and detection methods of RBC EVs. We also emphasized recent studies investigating the role of RBC EVs in various diseases, such as hypercoagulable states, Parkinson’s disease, cardiovascular disease, hematoma, sickle cell disease, and malaria. Furthermore, we discussed the potential application of RBC EVs as diagnostic and prognostic biomarkers in diseases, as well as their potential use as drug delivery vectors in the fight against various diseases. However, it is important to acknowledge that despite the many benefits that RBC EVs offer, there are several challenges that need to be addressed for their practical application, and these challenges are also highlighted in this review.

## 2. Biogenesis of RBC EVs

RBCs lack a nucleus and cannot synthesize lipids and proteins [16]. During their maturation into erythrocytes, reticulocytes specifically eliminate certain proteins, such as the transferrin receptor (TfR or CD71), and membrane-associated enzymes by forming multivesicular bodies (MVBs) [17]. Subsequently, the EVs within these MVBs are released into the extracellular space through fusion with the plasma membrane [16]. On average, a human RBC sheds approximately 20% of its membrane area throughout its lifespan. It has been proposed that in order for a young RBC with a surface area of 135 µm^2^ to transform into an old RBC with a surface area of 112 µm^2^, approximately 325 EVs with a diameter of 150 nm need to be lost [18]. If vesicles are lost linearly over time, an RBC will release an estimated 2.71 EVs per day during its 120-day lifespan [18]. According to a previous study, the shedding of EVs from RBCs is believed to be influenced by calcium rises and oxidative stress, although the exact mechanism behind this process remains unclear. It has been observed that RBC EVs are formed at regions of the cell membrane that do not contain cytoskeleton components. These cytoskeleton-free regions may play a role in facilitating the release of EVs from the RBC membrane [19]. Phosphatidylserine (PS) is typically present on the inner leaflets of cell membranes, but when it becomes exposed on the outer surface, it acts as a signal for phagocytosis and triggers the activation of coagulation. Therefore, the removal of externally exposed PS from RBCs through EVs may serve as a survival mechanism for these cells [20].

**Figure 1:**
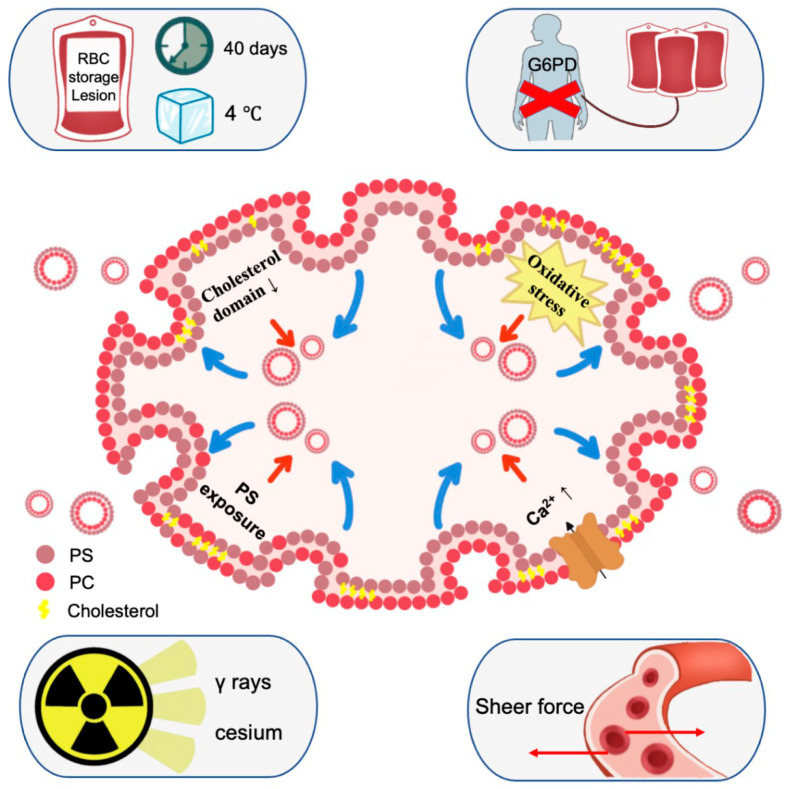
Biogenesis of red blood cell-derived EVs. Calcium rise and oxidative stress are the two main models for sEV shedding of RBC, while the specific mechanism remains completely unclear. There are probably four successive events during the biogenesis of RBC EVs: a. cholesterol domain decrease; b. oxidative stress; c. sphingomyelin/sphingomyelinase/ceramide/calcium alteration; d. phosphatidylserine exposure. The storage, deficient in glucose-6-phosphate dehydrogenase (G6PD), gamma rays, and shear rate are associated with increased biogenesis of RBC EVs.

When human RBCs are treated with agents that increase intracellular calcium levels (such as A23187, LPA, or PMA) in the presence of extracellular calcium, it leads to the release of EVs from the RBCs [21]. Cloos et al. proposed four successive events during the biogenesis of RBC EVs: (a) decrease in cholesterol domain, (b) oxidative stress, (c) alteration in sphingomyelin/sphingomyelinase/ceramide/calcium, and (d) exposure of phosphatidylserine (Figure 1) [22]. Vorselen et al. conducted a study to investigate the mechanical properties of EVs derived from RBCs. The study revealed that RBC EVs demonstrate mechanical behavior comparable to fluid liposomes. The bending modulus of RBC EVs derived from healthy donors was found to be approximately 15 Kb T, which is consistent with previously reported values for liposomes [23] and RBC membrane [24]. In the case of patients with hereditary spherocytosis, the RBC EVs exhibit an altered protein composition and a noticeably softer membrane compared to healthy individuals [24].

Red blood cell concentrates (RCCs) are commonly used as a transfusion therapy worldwide. It has been shown in studies that RBC EVs are present in RCCs. Importantly, the release of EVs is recognized as a characteristic of the RBC storage lesion [25]. Therefore, the absolute number of RBC EVs gradually increases with the duration of hypothermic storage. After 50 days of storage at 4 °C, a substantial 20-fold increase in the number of EVs was observed in RBC supernatants [26]. Studies have shown that RCCs produced using whole blood filtration have higher numbers of RBC EVs compared to RCCs produced using red cell filtration. Moreover, stored RCCs from blood donors who are deficient in G6PD exhibited a higher level of RBC EVs compared to G6PD-normal donors [12]. The results suggest that the G6PD status of blood donors is associated with the concentration of RBC EVs. Additionally, other studies have found that the release of EVs in RCCs is increased after exposure to gamma rays from a cesium-137 irradiator [27] or shear stress [28]. Higher concentrations of RBC EVs were observed in RBCs when subjected to increased shear rate and exposure time. This is because shear stress can lead to hemolysis or non-reversible sub-hemolytic damage to RBCs, and the concentration of RBC EVs may serve as an indicator of RBC shear rate-related damage. Furthermore, the contents of RBC EVs are correlated with measures of hemolysis and other RBC quality indicators, such as ATP levels and RBC deformability. Therefore, monitoring the contents of RBC EVs in stored RCCs holds promise as a non-destructive routine indicator of product quality for RCCs [29]. The size and concentration of RBC EVs in RBC concentrate products are influenced by blood manufacturing methods and the duration of hypothermic storage [30]. However, further studies are required to gain a better understanding of these heterogeneous populations of RBC EVs and their potential applications.

Due to its promising therapeutic properties, platelet-rich plasma (PRP) has been used in regenerative medicine for more than 30 years [31]. Undoubtedly, EVs are presented in PRP. The primary distinction and similarities between RBC EVs and PRP EVs lie in their distinct cellular origins. As the name implies, RBC EVs are secreted by RBCs [31]. Therefore, their cellular source is indeed very singular. On the other hand, PRP EVs typically refer to the EVs present in platelet-rich plasma (PRP), which includes various subtypes of circulating EVs derived from platelets, endothelial cells, RBCs, and leukocytes in the peripheral blood. PRP EVs also include RBC EVs.

## 3. Cargo Profiles of RBC EVs

Bebesi et al. employed attenuated total reflection infrared (ATR-IR) spectroscopy, a relatively simple and rapid method, to detect the presence of proteins, lipids, organophosphates, and carbohydrates in RBC EVs [19]. And they found that the characteristics of RBC EVs are determined by the storage conditions of RBCs. In 2018, Díaz-Varela et a. reported the first mass spectrometry-based proteomics of human reticulocyte-derived EVs [17]. The protein cargo profile of human reticulocyte-derived EVs comprises 367 proteins, with a majority of them being related to transporters, as well as proteins involved in EV biogenesis and erythrocytic disorders. Immunoelectron microscopy techniques confirmed the presence of the transferrin receptor. Huang et al. conducted a study on RBC EVs and reported that these EVs exhibited a higher abundance of short RNA compared to long RNA [32]. The top three miRNAs detected in RBC EVs were miR-125b-5p, miR-4454, and miR-451a. Moreover, the levels of miR-4454 and miR-451a were increased with the storage time of RBC. Damage-associated molecular patterns (DAMPs) and mitochondrial (mt) DNA were presented in RBC EVs, and their levels vary by manufacturing method [7]. A previous study compared the phospholipid composition of RBC and RBC EVs [16] and found that the phospholipids were largely similar between RBCs and RBC EVs. No accumulation of raft lipids was detected in EVs, suggesting that the biogenesis of RBC EVs during storage might not be raft-based. In addition to similarities, there are also differences in the composition of RBCs and RBC EVs. Wararat Chiangjong et al. recently summarized and compared the main components of RBC and RBC EVs [16].

In a recent study by Oh et al., it was reported that large extracellular vesicles (lEVs) and small extracellular vesicles (sEVs) derived from stored RBCs carry diverse cargoes and elicit distinct cellular effects [33]. Unbiased proteomics analyses of these vesicles revealed differential expression of 169 proteins. The most notable variation was observed in hemoglobin and heme content. Interestingly, the study findings indicated that oxidized proteins were preferentially packaged into EVs. Additionally, the total glutathione (GSH + GSSG) levels in sEVs were found to be lower than those in lEVs. Previous studies reported a higher sEV accumulation and proinflammatory activity in non-leukoreduced RBC units when compared to leukoreduced ones [26,34]. Tzounakas et al. further conducted a paired proteomics study on RBC units prepared by the same donors and stored from the same period with or without prestorage leukoreduction [34]. They revealed the distinct cargo profiles of RBC EVs under the two RBC preparation methods.

## 4. Detection of RBC EVs

In terms of protein markers, EVs are commonly characterized by the presence of CD9, CD63, and CD81. Other protein markers often associated with exosomes include flotillin, TSG101, Alix, HSP60, HSP70, HSPA5, CCT2, and HSP90 [35]. Additionally, each subset of EVs secreted by different cells possesses unique markers specific to their donor cells. CD235a, the surface marker of RBCs, was also found on RBC EVs and identified as a marker of RBC EVs [36]. Glycophorin A and B were identified on EVs derived from RBCs [37]. Carboxyfluorescein succinimidyl ester (CFSE), a commonly used dye for EVs, was used to identify RBC EVs with the assistance of glycophorin A. A more precise approach to indicate RBC EVs was observed when employing double staining with CFSE and glycophorin A, as compared to using CFSE or glycophorin A alone. This combined staining method provided enhanced accuracy in the identification and detection of RBC EVs [38]. Other molecules that were usually found on RBC EVs, such as Annexin V, have also been recommended for use as a marker for RBC EVs [39]. In addition to nanoparticle tracking analysis, the absolute counting of RBC EVs can be evaluated by a quantitative method called “flow rate-based assay using red cell bead” with low cost [40].

The characteristics of RBC EVs have been found to display good stability across a wide range of conditions [41]. Almizraq et al., on the other hand, conducted an assessment of the size and concentration of EVs in stored RBC products using various detection methods, including tunable resistive plus sensing, flow cytometer, and dynamic light scattering. Interestingly, the study revealed that the size and concentration of RBC EVs were influenced by the choice of detection methods [42]. These findings suggest that combining multiple detection methods could be crucial in improving the characterization and study of EVs in stored RBCs.

## 5. RBC EVs in Diseases

**Figure 2:**
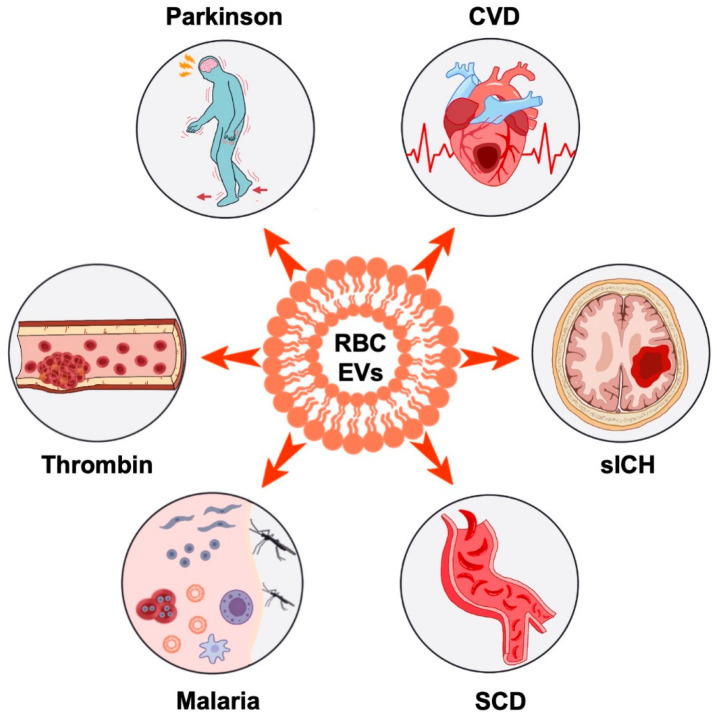
RBC EVs in diseases. RBC EVs are involved in various diseases, including hypercoagulable state, Parkinson’s disease, cardiovascular disease (CVD), Spontaneous intracerebral hemorrhage (sICH), sickle cell disease (SCD), malaria, and thrombin.

Multiple studies have provided evidence that RBC EVs play a role in various diseases, including but not limited to hypercoagulable state, Parkinson’s disease, cardiovascular disease (CVD), Spontaneous intracerebral hemorrhage (sICH), sickle cell disease (SCD), malaria, and thrombin (Figure 2).

### 5.1. Procoagulant Activity and Hemostasis

RBC EVs exhibit procoagulant properties due to the expression of tissue factor and/or a PS surface, which supports the assembly of enzymatic coagulation complexes [43]. Numerous studies have reported a significant hypercoagulation effect following the transfusion of stored RBCs [44]. In recent years, RBC EVs, whose amount accumulated during storage of RCCs, have been identified as involved in transfusion-related hypercoagulation [45]. The RBC EVs decelerated fibrin clot formation from fibrinogen in the presence of exogenous thrombin both with and without heparin [44,46], demonstrating the prominent antithrombin activity of RBC EVs. Moreover, the procoagulant activity of RBC EVs significantly increases during RCC storage [46]. The procoagulant activity of RBC EVs isolated from stored RCCs after 24 and 48 h storage was even different [47]. Although it is widely recognized that the storage of RBC is associated with impairment of their properties that can induce a circulatory risk to recipients, the specific mechanisms related to this phenomenon are still not fully understood currently. It is widely speculated that the composition components of RBCs and RBC EVs might undergo changes during the storage process [29,42]. These changes could potentially contribute to the observed enhanced proinflammatory host response, procoagulant activity, and hemostasis. For example, the increased level of PS may contribute to this phenomenon. However, further research is needed to fully elucidate the underlying mechanisms. Using healthy blood donor dogs, Avenick et al. reported that the procoagulant activity of the RBC EVs was due to their exposure to PS [48,49]. Kim et al. found that RBC EVs only induce a transient (few hours) hypercoagulable state in C57BL/6 mice [50]. RBC EVs had a prominent fibrinolytic activity, which cleaves fibrin [51].

The procoagulant activity of RBC EVs can be further enhanced by thermal trauma [52]. Because of their broad hemostatic activity, which enhances both primary (platelet) and secondary (coagulation) hemostasis, RBC EVs have been considered potential hemostatic agents for treating bleeding disorders [53]. For instance, RBC EVs could potentially be used as therapeutic agents to improve hemostatic defects in patients with platelet and coagulation disorders. The ability of RBC EVs to promote clotting may offer new avenues for developing treatments aimed at enhancing hemostasis in individuals with bleeding complications.

The roles of clotting factors in RBC EV-induced hypercoagulable state have been evaluated previously [50]. RBC EVs were unable to induce thrombin generation in the presence of corn trypsin inhibitor, a serine protease inhibitor that specifically inhibits the activation of human coagulation factor XII. This finding suggests that RBC EVs support factor XII-dependent thrombin generation [54]. Noubouossie et al. revealed RBC EVs activate both factor XII (FXII) and prekallikrein, leading to factor IX (FIX) activation by two independent pathways: the classic FXIIa-FXI-FIX pathway and direct kallikrein activation of FIX [43,55]. Rubin et al. also showed that RBC EVs have FXI-dependent procoagulant properties and can initiate TG in plasma in the absence of exogenous tissue factors [6]. They further revealed that the anionic surface of RBC EVs might be the site of FXI-mediated TG amplification and intrinsic tenase and prothrombinase complex assembly. Kim et al. revealed that RBC EV treatment increases microthrombi formation in lung vasculature in mice [56]. The presence of RBC EVs significantly increased the levels of P-selectin in cultured lung endothelial cells. Moreover, pre-treatment with P-selectin effectively reduced the formation of microthrombi in mice. On the other hand, Fischer et al. discovered that RBC EVs initiate coagulation through tissue factor signaling [57].

### 5.2. Transfusion-Related Adverse Effects

As a commonly used lifesaving method, blood transfusion has also been proposed to contribute to mortality, especially in critically ill patients [58]. The storage of RBCs would result in the accumulation of RBC EVs. And the high concentrations of RBC EVs in the stored RCCs were found to be related to adverse effects (e.g., thrombosis complications and transfusion-related acute lung injury) of blood transfusion [12,59]. The level of RBC EVs in human circulation was increased 2.4-fold in two hours after the transfusion of stored RCCs containing a high amount of RBC EVs. Then, the increased RBC EVs rapidly decreased within hours [60]. Previous studies revealed that the endogenous RBC EVs were cleared by macrophage phagocytosis, which mediated the recognition of membrane markers (e.g., PS, CD47). Surprisingly, these RBC membrane markers that are associated with macrophage clearance were hardly detected in EVs isolated from stored RBCs, indicating the distinct clearance mechanisms between the two kinds of RBC EVs [60]. Peters et al. also found that EVs from autologous stored RBC products could be detected in the recipient after transfusion [61]. And other studies demonstrated that EVs accumulated during the storage of RBCs and stimulated thrombin generation in vitro. Stored RBC-derived EVs cause lung injury after hemorrhage and resuscitation by increasing pulmonary neutrophil accumulation [62,63]. CD11 expression, superoxide production, and phagocytic ability of neutrophils were enhanced by EVs isolated from stored RBCs. However, Peters et al. revealed that transfusion of autologous EVs from one unit of stored RBCs does not augment thrombin generation in a model of human endotoxemia induced by LPS injection [61].

Adherence of blood cells to the microvasculature may result in RBC transfusion-induced organ failure. Stored RBC-derived EVs were phagocytosed by monocytes through CR3 and activated monocytes with subsequent upregulation of endothelial cell adhesion markers [64]. This suggested that stored RBC-derived EVs induce a proinflammatory and procoagulant endothelial cell response. The immunomodulatory effects of EVs in stored RCCs have attracted much attention in transfusion medicine. Stored RBC-derived EVs exhibited a negative role in B cell survival, plasmacytic differentiation, and response to LPS stimulation [65], indicating their roles in transfusion-related immunomodulation, a prominent complication of blood transfusion.

### 5.3. Parkinson’s Disease (PD)

As a common neurodegenerative disorder, Parkinson’s disease (PD) is characterized by neuronal death in multiple brain regions, and pathophysiology develops in part from the formation, transmission, and aggregation of toxic species of the protein α-synuclein (α-syn) [66]. Recent studies have uncovered the involvement of EVs in the transportation of toxic α-syn between different brain regions, thereby contributing to the development of PD [67]. As a source of potentially pathogenic α-syn, RBCs contain α-syn concentrations ~1000-fold higher than the cerebrospinal fluid. There is no surprise that α-syn was presented in RBC EVs. Matsumoto et al. found that RBC produces α-syn-rich EVs, which can cross the blood–brain barrier and be untaken by microglial cells, promoting microglial inflammatory responses [67]. Inflammation, including hyperactivation of monocytes in the central nervous system, is another salient feature of PD pathogenesis. Liu et al. reported that α-syn-enriched RBC EVs isolated from PD model mice induced the inflammatory sensitization of THP-1 cells in an endocytosis-dependent manner [68]. Additionally, leucine-rich repeat kinase 2 (LRRK2), another key protein involved in PD, was involved in this pathological process.

### 5.4. Cardiovascular Disease (CVD)

An increasing number of studies suggest that RBC EVs, which have procoagulant and vasoconstrictive effects, are involved in the development of acute cardiovascular events. Yuan et al. revealed increased levels of RBC EVs in Chinese individuals with acute myocardial infarction compared to those with non-coronary artery disease. However, no significant difference in RBC EV levels was observed in Thrombolysis in Myocardial Infarction (TIMI) risk stratification [69]. Giannopoulos et al. reported that STEMI patients treated with primary percutaneous coronary intervention (PCI) had increased levels of RBC EVs (twice that of healthy volunteers). Furthermore, a high level of RBC EVs appears to be positively associated with adverse clinical events following primary angioplasty [70]. Valkov et al. [71] used a transgenic mouse model to study the recipient cells of RBC EVs in vivo. Functional Cre mRNA was packaged into EVs by Cre recombinase-expressing RBC cells. Then, these EVs transfer functional Cre to EV target cells, resulting in Cre-mediated recombination and expression of mGFP in the target cells. mGFP were detected in cells in various organs, including the heart, kidney, lungs, spleen, and brain. They specifically studied the role of RBC EVs in ischemic heart failure using single-cell nuclear RNA sequencing and revealed the complex cellular network of RBC EV-mediated intercellular communication. Functionally, the analysis showed that RBC EVs promoted post-infarct cardiac remodeling by altering the transcriptional profiles of cardiomyocytes and enhancing DNA synthesis.

Atrial fibrillation (AF) and heart failure (HF) are common cardiovascular (CV) conditions, which frequently complicate type 2 diabetes mellitus (T2DM) and exert a combined detrimental impact on CV mortality [72]. The level of RBC EVs in the AF group was significantly higher than that of the non-AF group. And the level of RBC EVs in patients with T2DM and HF was nearly twofold to that of individuals with T2DM but not HF. The number of RBC EVs in T2DM patients with HF depends on glycemia control; a high level of glycosilated hemoglobin indicates a high amount of RBC EVs in circulation [73]. Despite the underlying molecular mechanism remaining unknown, the elevated levels of RBC EVs may be used as an independent predictor for poor glycemia control in T2DM patients with HF and AF. Hematoma of Spontaneous intracerebral hemorrhage (sICH) is a disabling stroke sub-type with no effective therapies that is responsible for as much as 28% of stroke cases worldwide [74]. The size of the hematoma will expand for several hours after symptom onset, and the increased size of the hemorrhage often portends a poor prognosis, including mortality. Therefore, limiting hematoma expansion is a promising, effective therapeutic strategy for sICH. Recently, Rehni et al. reported that, as new and potent hemostatic agents, RBC sEV treatment 4.5 h post-sICH limited hematoma volume (reduced by 24%) and neurological impairment in a rat model [75]. The difference was, however, not significant when RBC EVs were administrated 6 h after sICH, indicating that RBC EVs appear to have a therapeutic effect window. In another study, they further determined the optimal dose, dosing regimen, and therapeutic time window of RBC EVs, which are critical to therapeutic outcomes in the rat model [76]. More importantly, RBC EV treatment also improved long-term histopathologic and behavioral outcomes of sICH.

### 5.5. Sickle Cell Disease (SCD)

Sickle cell disease (SCD) is a genetically inherited blood disorder in which some RBCs are shaped like sickles or crescent moons [77]. These sickle cells become rigid and sticky and are prone to extra- and intravascular hemolysis. A blood and bone marrow transplant is currently the only cure for sickle cell disease. Endothelial activation and sickle RBC adhesion are central to the pathogenesis of SCD. Compared with healthy RBCs, sickle RBCs in SCD patients in studies showed an increased ability to secrete EVs, which was further enhanced when the patients were in crisis [78,79]. This may result from, at least in part, the increased oxidative stress in SCD patients, which could promote the release of RBC EVs [80]. And sickle RBC EVs promote endothelial cell activation through cell signaling and transcriptional regulation [81]. Compared with healthy control, sickle RBC EVs promoted human pulmonary microvascular endothelial cell (HPMEC) activation, indicating increased von Willebrand factor (VWF) expression. RBC EVs isolated directly from SCD patient plasma showed increased adhesion molecule expression and the production of cytokines by human aortic endothelial cells (HAECs) compared to those isolated from the plasma of healthy individuals [80]. This suggested that RBC EVs play a role in vascular dysfunction in SCD. Further study revealed that RBC sEV-mediated endothelial cell activation was alleviated by TLR4 inhibition, demonstrating that TLR4 was involved in the processes. The level of RBC EVs in SCD patients was associated with hemolysis, the typical manifestation of SCD [82]. Additionally, the removal of RBC EVs from SCD patient plasma samples resulted in a seven-fold increase in clotting time, demonstrating that RBC EVs may contribute to the hypercoagulable state of SCD patients.

### 5.6. Malaria

Malaria remains one of the greatest public health challenges worldwide. Studies have identified the roles of plasmodium falciparum-infected RBC-derived EVs in the pathogenesis, activation, and modulation of host immune responses and play critical roles in the pathological process of malaria [83]. The level of RBC EVs in mice was elevated during infection and decreased rapidly after antimalarial treatment [84]. The EV secretion amount of plasmodium falciparum-infected RBCs was 10-fold higher than non-infected RBCs and increased as the parasites matured [8]. Babatunde et al. isolated EVs from malaria-infected RBCs and evaluated the RNA profiles by RNA-seq [85]. While the miRNAs and tRNA were the most abundant human RNAs in EVs released by malaria-infected RBCs, they also found Y-RNAs, vault RNAs, snoRNAs, and piRNAs. Furthermore, plasmodium RNAs were presented in these EVs and could be transferred to human endothelial cells, indicating their potential role of malaria-infected RBC EVs RNAs in regulation and cellular communication. Mantel et al. found that miRNAs (e.g., miR-451a) in EVs released by malaria-infected RBCs [86] form a functional complex with argonaute 2. And these complexes were transferred into endothelial cells by malaria-infected RBC-derived EVs, specifically silencing gene expression in endothelial cells and altering their barrier properties in a dose- and time-dependent manner. In addition to RNAs, plasmodium proteins, including the ring-infected erythrocyte surface antigen (RESA), were also detected in EVs from malaria-infected RBCs [85]. Moreover, these plasmodium RNAs and proteins containing EVs have proinflammatory activity and induce host inflammatory and immune responses (activate host monocytes and neutrophils), contributing to pathology during malaria infection [87].

Natural killer (NK) cells provide the first line of defense against malaria parasite infection. A study revealed that EVs released by malaria-infected RBCs activate NK cells via the RIG-I-like receptor pathway (MAD5) [88]. Monocytes are the primary immune cells to eliminate malaria-infected RBCs. Primary human monocytes that were stimulated with malaria-infected RBC-derived EVs released lower levels of inflammatory cytokines and showed transcriptomic changes [89]. And PfEMP1 presented in malaria-infected RBC-derived EVs was the critical mediator of this process. Another study identified another P. falciparum protein, PfPTP2, that plays a key role in efficient cell–cell communication between malaria-infected RBCs [90], suggesting that different biomolecules were involved in pathological processes in different receipt cells. Khowawisetsut et al. revealed that EVs derived from plasmodium falciparum-infected RBCs promoted the M2 polarization of monocytes [91], while the underlying mechanisms remain unclear.

## 6. The Applications of RBC EVs

### 6.1. RBC EVs as a Diagnostic and Prognostic Biomarker in Diseases

Infection of various susceptible cells by dengue virus (DENV) led to apoptotic death and release of EVs, which harbored a viral envelope protein and a nonstructural protein 1 (NS1) on their surfaces. Punyadee et al. revealed that elevated levels of RBC EVs, the major populations in the circulation of DENV-infected patients, directly correlated with DENV disease severity. Thus, RBC EVs have been put forward as a potential biomarker for dengue virus infections [10].

The number of RBC EVs in urinary patients with glomerular hematuria (GH) was significantly higher than that of non-glomerular hematuria (NGH), providing a predictive tool for classifying GH in the future [92]. The amounts of PS-positive RBC EVs were significantly increased in pulmonary arterial hypertension (PAH) patients when compared with normal subjects [93]. The concentration of RBC EVs (26/μL) in patients with type 2 diabetes mellitus was higher than that of patients with non-type 2 diabetes mellitus (9/μL). Moreover, the level of RBC EVs, which could be used to predict the presence of type 2 diabetes mellitus, was positively associated with fasting blood glucose but not with glycated hemoglobin [94]. The increased level of total RBC EVs and PS-positive RBC EVs were presented in patients with systemic lupus erythematosus (SLE), while there is no association with disease activity [95]. The high level of PS-positive RBC EVs indicated the high incidence of past thrombotic events [95].

The concentration of circulating EVs, which were largely derived from RBCs (45%) and platelets (30%), was significantly increased in patients with G6PD-deficient patients than healthy donors (1051/μL vs. 258/μL) [9]. Among G6PD-deficient patients, the concentration of circulating EVs in the severe G6PD deficiency group was significantly higher than in the moderate group (2567/μL vs. 984/μL). Thus, the concentration of circulating EVs could discriminate the disease stages of G6PD deficiency.

### 6.2. RBC EVs as Drug Delivery Vectors

As the natural delivery tool, RBC EVs possess many more favorable properties than other delivery vesicles, including liposomes. Briefly, when compared to other liposome-based drug delivery technologies, the use of red blood cell (RBC)-derived EVs offers several advantages: (i) Using blood donations to produce RBC-derived EVs is the readily available source of RBCs from existing blood banks. This eliminates the need for in vitro cell culture and the associated risks of genetic mutations or the requirements for cGMP-qualified media and supplements. (ii) Large-scale amounts (1013–1014) of EVs can be purified from RBCs after the treatment with calcium ionophore, thus providing a scalable strategy to obtain EVs. (iii) RBC EVs are safe. Unlike other cell types that contain a nucleus with DNA, RBCs naturally lack a nucleus during their maturation process. This means that RBC-derived EVs derived from enucleated RBCs are devoid of genetic material, including DNA [13,16]. This characteristic ensures that the contents of RBC-derived EVs remain homogeneous and do not carry the risk of introducing foreign genetic material or causing unpredictable effects.

Before they were developed into drug delivery vectors, the security of RBC EVs was a primary consideration. Previous retrospective studies suggested that RBC transfusion was associated with poor progression with increased rates of cancer recurrence, metastasis, and death in patients with colorectal cancer [11]. To evaluate the role of RBC EVs in RBC transfusion-related poor progression, Fischer et al. systematically evaluated the in vitro effect of RBC EVs on colorectal cancer cells [11]. They showed that RBC EVs did not affect functional and phenotypic characteristics of different colon carcinoma cells, exerting no cancer-promoting effects. Rehni et al. evaluated the safety and biodistribution in vivo using male rat models [96]. No significant changes in the physiological parameters (e.g., blood pressure, body and head temperature, hematocrit, and blood gases) were observed in sixty mice after injection of RBC EVs, indicating the good biocompatibility of RBC EVs. However, since the post-injection observation period was relatively short, long-term studies confirming the safety of RBC EVs are warranted.

RBC EVs were developed as delivery vectors of RNA (including antisense oligonucleotides, Cas9 mRNA, and guide RNAs), siRNA, and immRNA to fight against various diseases, including cancer [2,3,4]. Long lifetimes and strong tissue-specific targeting are essential for RBC EVs to deliver drugs more effectively. Previous studies have revealed the distinct kinetics of circulating EV subsets with different cell sources. RBC EVs showed the longest circulation time due to the high level of “don’t eat me” signal CD47, which prevents phagocytosis by macrophages [97]. The lifetime of RBC EVs could be further prolonged by membrane cholesterol enrichment, which reduces PS externalization on the surface [98]. LTH peptide, which could bind to the kidney injury molecule-1 (Kim-1), was modified on the surface of RBC EVs to improve their kidney tissue-specific targeting [14] in acute kidney injury (AKI) models. The expression of epidermal growth factor receptor (EGFR), a regulator of cell growth in tissues of epithelial origin, is often elevated in various cancers. Conjugation of RBC EVs with EGFR-binding nanobodies promotes specific delivery of RNA to metastatic breast cancer cells [99].

Borgheti-Card et al. developed targeted antimalarial drug delivery vehicles using EVs derived from plasmodium-infected and non-infected RBCs [100]. Compared with the free hydrophobic drug counterparts, the antimalarial drugs wrapped in RBC EVs showed increased efficiency in inhibiting P. falciparum growth in vitro.

## 7. Current Challenges and Opportunities

### 7.1. Selective Purification of RBC EVs

**Figure 3:**
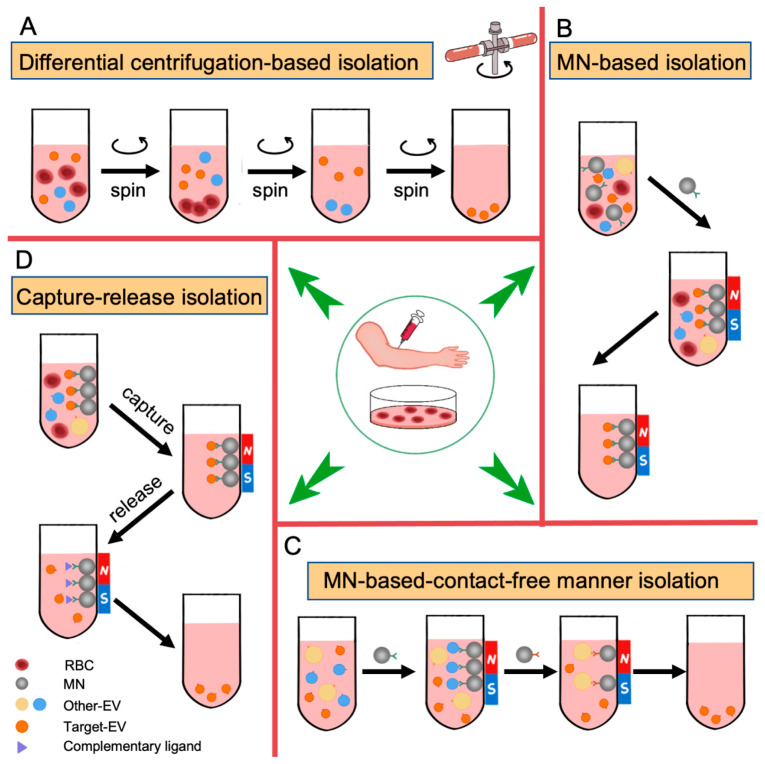
The isolation methods of RBC EVs. Isolation methods of sEVs were divided into differential centrifugation-based isolation (**A**), MN-based isolation (**B**), MN-based contact-free manner isolation (**C**), and capture–release isolation (**D**).

Given that RBC EVs are often mixed with EVs released by other cell types in body fluids, it is crucial to selectively isolate RBC EVs in order to study their unique cargo profiles and biological effects. However, the current gold standard method for isolating EVs, ultrahigh-speed differential centrifugation, fails to effectively purify RBC EVs. Because EVs released by different cell sources possess distinct surface markers, surface marker-based isolation methods, such as using CD235 antibody-functionalized magnetic nanoparticles, appear to be more appropriate for the specific purification of RBC EVs. However, one challenge with this approach is the difficulty in removing the magnetic nanoparticles from the purified RBC EVs. Although these large and heavy magnetic nanoparticles do not adversely affect subsequent analysis of EV cargo, they can impede functional studies of RBC EVs. Recently, contact-free capture–release methods or other isolation strategies have been reported for the selective purification of EVs with specific surface markers [36,101]. These innovative techniques effectively preserve the biological functions and structural integrity of the isolated EVs, making them suitable for the selective purification of RBC EVs (Figure 3). These advances offer promising avenues for investigating the properties and roles of RBC EVs.

### 7.2. The Heterogeneity of RBC EVs

The secretion of EVs by RBCs is an ongoing process throughout their long lifespan, which can extend up to 120 days. RBCs of different ages exhibit varying cargo profiles, such as differences in the level of CD47. As a result, EVs released by these heterogeneous RBCs display distinct cargo profiles. However, there have been very few studies that have explored the heterogeneity of RBC EVs until now. Undoubtedly, uncovering the heterogeneity of RBC EVs is essential for conducting detailed and in-depth research on the biological behaviors and critical roles of RBC EVs in the future.

### 7.3. Application of RBC EVs in Stored RBC Products: Eliminate Them or Exploit Them

RBC EVs present in stored RBC products have been implicated in transfusion-related adverse effects. As mentioned earlier, RBC EVs in stored RBC products demonstrate a significant procoagulant effect, which is unfavorable and may worsen the condition of patients with a hypercoagulable state. Hence, to minimize transfusion-related adverse effects, it seems imperative to remove RBC EVs from stored RBC units before transfusion. However, the challenge lies in finding effective methods to eliminate these EVs from RBC units, which is a complex problem that requires further research. On the other hand, the procoagulant activity of RBC EVs can be exploited for their potential use as hemostatic agents in the treatment of bleeding. Exploring alternate applications for RBC EVs could lead to new scenarios where their value in stored RBC units can be maximized. Further studies are needed in the future to uncover these possibilities and optimize the utilization of RBC EVs.

### 7.4. The Legal Framework of Using RBC EVs

Currently, there is no specific legal framework governing the use of blood donations for the production of RBC EVs. However, considering that RBC EVs are considered a component of blood or tissue products, it is anticipated that the legal framework for the use of RBC EVs will eventually align with that of other blood or tissue products. Consequently, the collection of RBC EVs would most likely be a volunteer-based endeavor, and as such, commercialization of RBC EV products with a specific price is unlikely in the near future.

## 8. Conclusions

In summary, our present review comprehensively reviewed the current research status of RBC EVs, especially emphasizing their biological functions in various diseases. Moreover, this review also discussed their application as a diagnostic and prognostic biomarker in diseases and drug delivery vectors. Importantly, we highlighted several potential changes and solutions based on our understanding. Further effort is needed to realize the selective purification of RBC EVs and uncover their heterogeneity.

## Data Availability

Not applicable.
